# Inositol 1,4,5‐trisphosphate receptors and their protein partners as signalling hubs

**DOI:** 10.1113/JP271139

**Published:** 2016-02-24

**Authors:** David L. Prole, Colin W. Taylor

**Affiliations:** ^1^ Department of PharmacologyUniversity of CambridgeCambridgeCB2 1PDUK

## Abstract

Inositol 1,4,5‐trisphosphate receptors (IP_3_Rs) are expressed in nearly all animal cells, where they mediate the release of Ca^2+^ from intracellular stores. The complex spatial and temporal organization of the ensuing intracellular Ca^2+^ signals allows selective regulation of diverse physiological responses. Interactions of IP_3_Rs with other proteins contribute to the specificity and speed of Ca^2+^ signalling pathways, and to their capacity to integrate information from other signalling pathways. In this review, we provide a comprehensive survey of the proteins proposed to interact with IP_3_Rs and the functional effects that these interactions produce. Interacting proteins can determine the activity of IP_3_Rs, facilitate their regulation by multiple signalling pathways and direct the Ca^2+^ that they release to specific targets. We suggest that IP_3_Rs function as signalling hubs through which diverse inputs are processed and then emerge as cytosolic Ca^2+^ signals.

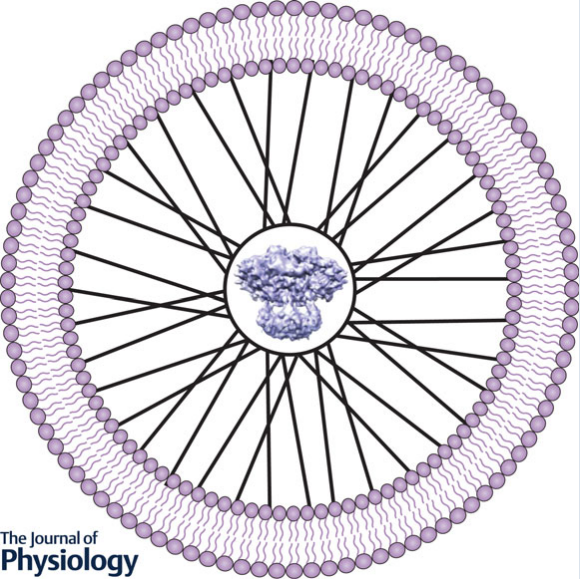

**Abbreviations**
AC: adenylyl cyclase
B_2_R: type 2 bradykinin receptor
cAMP: cyclic adenosine monophosphate
CREB: cAMP response element‐binding protein
EB3: end‐binding protein 3
ER: endoplasmic reticulum
GPCR: G protein‐coupled receptor
IBC: IP_3_‐binding core
IP_3_: inositol 1,4,5‐trisphosphate
IP_3_R: IP_3_ receptor
IRBIT: IP_3_R‐binding protein released with IP_3_
M_1_R: type 1 muscarinic acetylcholine receptor
PKA: protein kinase A
PLC: phospholipase C
SD: suppressor domain
TMD: transmembrane domain

## Introduction

Ca^2+^ signals within cells are spatially and temporally intricate, allowing them to elicit a multitude of specific downstream effects (Berridge, 2009). Inositol 1,4,5‐trisphosphate receptors (IP_3_Rs), the most widely expressed class of intracellular Ca^2+^ channel, release Ca^2+^ from intracellular stores in response to binding of IP_3_ and Ca^2+^ (Foskett *et al*. 2007; Taylor & Tovey, 2010). Dual regulation of IP_3_Rs by two essential stimuli, IP_3_ and Ca^2+^, is important because it endows IP_3_Rs with a capacity to propagate Ca^2+^ signals regeneratively by Ca^2+^‐induced Ca^2+^ release, as Ca^2+^ released by an active IP_3_R ignites the activity of adjacent IP_3_Rs that have bound IP_3_ (Smith & Parker, 2009). This in turn plays a key role in defining the spatial organization of IP_3_‐evoked Ca^2+^ signals.

**Figure 1:**
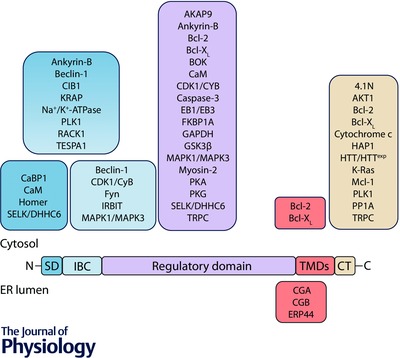
**Association of proteins with IP_3_Rs** Key functional domains of a single IP_3_R subunit are shown: the suppressor domain (SD), IP_3_‐binding core (IBC), cytosolic regulatory domain, transmembrane domains (TMDs) and the cytosolic C‐terminus (CT). The sites to which proteins are proposed to bind are shown. Many additional proteins are thought to associate with IP_3_Rs, but the binding sites have not been identified. Abbreviations and references are provided in Tables 1, 2, 3, 4.

Activation of IP_3_Rs is initiated by binding of IP_3_ within a clam‐like structure, the IP_3_‐binding core (IBC) (Bosanac *et al*. 2002), located near the N‐terminus of each IP_3_R subunit. Binding of IP_3_ causes a conformational change that rearranges the association of the IBC with the N‐terminal suppressor domain (SD). These changes are proposed to disrupt interactions between the N‐terminal regions of the four subunits of the IP_3_R, leading to opening of the channel. The latter is formed by transmembrane domains (TMDs) towards the C‐terminus of each IP_3_R subunit (Seo *et al*. 2012) (Fig. 1). It is not yet clear where binding of Ca^2+^ to the IP_3_R lies within the sequence of events linking binding of IP_3_ to channel gating. One possibility is that the conformational changes evoked by binding of IP_3_ expose a site to which Ca^2+^ must bind before the channel can open (Marchant & Taylor, 1997; Foskett *et al*. 2007). However, neither the structural identity of this stimulatory Ca^2+^‐binding site, nor that of the inhibitory site through which higher concentrations of Ca^2+^ inhibit IP_3_Rs have been resolved. The inhibitory site may reside on an accessory protein associated with IP_3_Rs.

IP_3_Rs are present in almost all animal cells and some protozoa (Prole & Taylor, 2011), but there are no homologous proteins in plants (Wheeler & Brownlee, 2008) or fungi (Prole & Taylor, 2012). The genomes of vertebrates encode three subtypes of IP_3_R subunit (IP_3_R1–3), which can form homo‐tetrameric or hetero‐tetrameric channels (Joseph *et al*. 1995) with differing properties and distributions (Foskett *et al*. 2007; Mikoshiba, 2007). In mammalian cells, IP_3_Rs have been reported to release Ca^2+^ mainly from the endoplasmic reticulum (ER) (Streb *et al*. 1984; Volpe *et al*. 1985), but the Golgi apparatus (Pinton *et al*. 1998) and secretory vesicles (Yoo, 2011) also respond to IP_3_. Although IP_3_ initiates Ca^2+^ signals by stimulating Ca^2+^ release from intracellular stores, the signals are sustained by Ca^2+^ entry across the plasma membrane. That too is indirectly regulated by IP_3_, because store‐operated Ca^2+^ entry is stimulated by loss of Ca^2+^ from the ER (Parekh & Putney, 2005; Lewis, 2012). Ca^2+^ signals initiated by IP_3_Rs evoke a wide variety of cellular events, ranging from embryological development (Kume *et al*. 1997; Uchida *et al*. 2010) to cellular metabolism (Cardenas *et al*. 2010), gluconeogenesis (Wang *et al*. 2012), exocrine secretion (Futatsugi *et al*. 2005) and neuronal function (Matsumoto *et al*. 1996).

Specificity within Ca^2+^ signalling pathways, or indeed any signalling pathway (Scott & Pawson, 2009; Scott *et al*. 2013), is achieved, in part, by the formation of macromolecular signalling complexes. Within the signalling pathways that involve phospholipase C (PLC), these complexes regulate the activity of IP_3_Rs, their distribution, and their association with both the plasma membrane receptors that evoke IP_3_ formation and the downstream targets of the Ca^2+^ released by IP_3_Rs (Konieczny *et al*. 2012). The interactions of IP_3_Rs with other proteins have been reviewed previously (Choe & Ehrlich, 2006; Foskett *et al*. 2007; Mikoshiba, 2007; Vanderheyden *et al*. 2009 *a*), but continued progress and the advent of high‐throughput proteomics methods (Havugimana *et al*. 2012; Rolland *et al*. 2014) suggest that an update is timely.

Searches of proteomic databases and published literature reveal a large number of proteins that form complexes with IP_3_Rs (Tables 1, 2, 3, 4). For some of these proteins, the regions within IP_3_Rs that are important for the interaction have been mapped (Fig. 1). At the outset, it is worth sounding some notes of caution regarding the reported interactions. Firstly, it is often difficult to establish that two proteins interact directly, rather than via intermediate proteins. Many of these complexes may, therefore, be formed by direct or indirect interactions of IP_3_Rs with other proteins. For example, association of protein phosphatase 1 with IP_3_Rs may be mediated in part by IRBIT (IP_3_R‐binding protein released with IP_3_), which binds directly to both proteins (Ando *et al*. 2014). Secondly, the interactions and their effects may depend on the cellular context, including such factors as the subtype of IP_3_R, the physiological status of the IP_3_R (e.g. phosphorylation), the cell type and the expression levels of the interacting proteins and IP_3_Rs. Thirdly, interactions that occur in cellular lysates may be precluded within intact cells. For example, the interaction of two proteins may be prevented by their physical separation within the cell or by mutually exclusive binding of other proteins or ligands. IRBIT, for example, binds to IP_3_R subunits only when they have no IP_3_ bound. Lastly, some forms of experimental evidence are more discriminating than others, and it will be necessary to verify the putative interactions indicated by methods such as yeast two‐hybrid screening and mass spectrometry.

**Table 1:** Proteins that form complexes with IP_3_Rs and enhance their activity

Protein	References
Effective delivery of messengers	
Adenylyl cyclase 6 (AC6)	Tovey *et al*. 2008
Bradykinin receptor B_2_ (B_2_R)	Delmas *et al*. 2002; Jin *et al*. 2013
Epidermal growth factor receptor (EGFR)	Hur *et al*. 2005
Erythropoietin receptor (EPO‐R)	Tong *et al*. 2004
Glyceraldehyde‐3‐phosphate dehydrogenase (GAPDH)	Patterson *et al*. 2005
Metabotropic glutamate receptor 1 (mGluR1;GRM1)	Tu *et al*. 1998
Phospholipase C‐β1 (PLCβ1)	Shin *et al*. 2000
Phospholipase C‐β4 (PLCβ4)	Nakamura *et al*. 2004
Phospholipase C‐γ1 (PLCγ1)	Tong *et al*. 2004; Yuan *et al*. 2005
Protease‐activated receptor 2 (PAR‐2)	Jin *et al*. 2013
Sensitization to IP_3_/Ca^2^ ***^+^***	
Bcl‐2 (B‐cell lymphoma 2)^a^	Chen *et al*. 2004; Eckenrode *et al*. 2010; Monaco *et al*. 2012; Chang *et al*. 2014
Bcl‐X_L_ (B‐cell lymphoma extra large)	White *et al*. 2005; Eckenrode *et al*. 2010; Monaco *et al*. 2012
Chromogranin A (CGA)	Yoo & Lewis, 1998; Thrower *et al*. 2002
Chromogranin B (CGB; secretogranin‐1)	Yoo & Lewis, 2000; Thrower *et al*. 2003
Cyclin‐A	Soghoian *et al*. 2005
Cyclin‐B1 (CYB)	Malathi *et al*. 2003; Malathi *et al*. 2005
Cyclin‐dependent kinase 1 (CDK1)	Malathi *et al*. 2003; Malathi *et al*. 2005
Cytochrome *c*_1_	Boehning *et al*. 2004
Fyn (tyrosine‐protein kinase)	Jayaraman *et al*. 1996; Cui *et al*. 2004
Glucosidase 2 subunit β (80K‐H)	Kawaai *et al*. 2009
Glycogen synthase kinase‐3β (GSK3β)	Gomez *et al*. 2016
Huntingtin‐associated protein 1 (HAP‐1)	Tang *et al*. 2003 *b*
Huntingtin (HTT) (with poly‐Q expansion, HTT^exp^)^b^	Tang *et al*. 2003 *b*
Lyn (tyrosine‐protein kinase)	Yokoyama *et al*. 2002
Mcl‐1 (myeloid cell leukemia‐1)	Eckenrode *et al*. 2010
mTOR (mammalian target of rapamycin)	Fregeau *et al*. 2011
Neuronal Ca^2+^ sensor 1 (NCS‐1)	Schlecker *et al*. 2006
Polo‐like kinase 1 (PLK1)	Ito *et al*. 2008; Vanderheyden *et al*. 2009 *b*
Presenilin‐1/Presenilin‐2 (PS‐1/PS‐2)	Cheung *et al*. 2008
Protein kinase A (PKA; cAMP‐dependent protein kinase)	Ferris *et al*. 1991; Bruce *et al*. 2002
Receptor of activated protein kinase C1 (RACK1)	Patterson *et al*. 2004
Rho‐associated protein kinase (ROCK)	Singleton & Bourguignon, 2002
TRISK 32 (cardiac triadin TRISK 32 isoform)	Olah *et al*. 2011
Direct activation of IP_3_Rs	
Ca^2+^‐binding protein 1 (CaBP1)^c^	Yang *et al*. 2002; Li *et al*. 2013
CIB1 (Ca^2+^ and integrin‐binding protein 1; calmyrin)^c^	White *et al*. 2006
Gβγ complex	Shin *et al*. 2000; Zeng *et al*. 2003
Other	
DARPP‐32 (protein phosphatase 1 regulatory subunit 1B)	Chang *et al*. 2014
DHHC6	Fredericks *et al*. 2014
EB3 (end‐binding protein 3)	Geyer *et al*. 2015
GRP‐78 (78 kDa glucose‐regulated protein; BiP)	Higo *et al*. 2010
Phosphatidylinositol trisphosphate 3‐phosphatase (PTEN)	Bononi *et al*. 2013
Selenoprotein K (SELK)	Fredericks *et al*. 2014

Data for Tables 1, 2, 3, 4 were derived from manual searches of the literature, reviews (Choe & Ehrlich, 2006; Foskett *et al*. 2007; Mikoshiba, 2007; Vanderheyden *et al*. 2009 *a*) and databases, including BioGRID (Chatr‐Aryamontri *et al*. 2015) and IntAct (Orchard *et al*. 2013). The nomenclature of proteins shown is consistent with the human homologues, although some data are derived from interactions of IP_3_Rs and proteins from other species. ^a^Some studies report sensitization of IP_3_Rs by Bcl‐2, while others report inhibition. ^b^HTT^exp^, but not wild‐type HTT, sensitizes IP_3_Rs to IP_3_/Ca^2+^. ^c^CaBP1 and CIB1 are also reported to inhibit IP_3_Rs (see Table 2); direct activation seems to occur only transiently, and is controversial.

**Table 2:** Proteins that form complexes with IP_3_Rs and inhibit their activity

Protein	References
Proteins that bind reversibly and disrupt activation by IP_3_ and/or Ca^2+^	
Ankyrin‐R (ANK1)	Bourguignon *et al*. 1993; Joseph & Samanta, 1993
Bcl‐2 (B‐cell lymphoma 2)^a^	Chen *et al*. 2004; Monaco *et al*. 2012; Chang *et al*. 2014
Ca^2+^‐binding protein 1 (CaBP1)^b^	Yang *et al*. 2002; Li *et al*. 2013
Calmodulin (CaM)	Maeda *et al*. 1991; Yamada *et al*. 1995
Carbonic anhydrase‐related protein (CARP; CA8)	Hirota *et al*. 2003
Caspase‐3	Hirota *et al*. 1999
CIB1 (Ca^2+^ and integrin‐binding protein 1; calmyrin)^b^	White *et al*. 2006
DANGER (IP_3_R‐interacting protein)	van Rossum *et al*. 2006
ERp44 (endoplasmic reticulum resident protein 44)	Higo *et al*. 2005
FKBP1A (FK506‐binding protein 1A; FKBP12)	Cameron *et al*. 1995 *b*
GIT1/GIT2 (ARF GTPase‐activating protein 1/2)	Zhang *et al*. 2009
IRBIT (IP_3_‐binding protein released with IP_3_)	Ando *et al*. 2003
K‐Ras	Sung *et al*. 2013
MRVI1 (IRAG; IP_3_R‐associated cGMP kinase substrate)	Schlossman *et al*. 2000
Nuclear protein localization protein 4 homologue (NPL4)	Alzayady *et al*. 2005
Polycystin‐1 (PC1; TRPP1)	Li *et al*. 2005
Proteins that post‐translationally modify IP_3_Rs	
AKT1 (RAC‐α serine/threonine protein kinase; PKB)	Khan *et al*. 2006; Szado *et al*. 2008
Ca^2+^/calmodulin‐dependent protein kinase II (CaMKII)	Ferris *et al*. 1991; Bare *et al*. 2005
Calpain	Μagnusson *et al*. 1993; Wojcikiewicz & Oberdorf, 1996
E3 ubiquitin ligase AMFR (GP78)^c^	Pearce *et al*. 2007
E3 ubiquitin ligase RNF170^c^	Lu *et al*. 2011
Erlin‐1/Erlin‐2 (SPFH domain‐containing protein 1/2)^c^	Pearce *et al*. 2007; Pearce *et al*. 2009
MAPK1/MAPK3 (mitogen‐activated protein kinase 1/3)	Bai *et al*. 2006
Protein phosphatase 1A (PP1A)	Tang *et al*. 2003 *a*; Chang *et al*. 2014
Transglutaminase‐2 (TGM2)	Hamada *et al*. 2014
Transitional endoplasmic reticulum ATPase (p97)^c^	Alzayady *et al*. 2005
Ubiquitin^c^	Bokkala & Joseph, 1997; Oberdorf *et al*. 1999
Ubiquitin‐conjugating enzyme E2 7 (UBC7)^c^	Webster *et al*. 2003
Ubiquitin conjugation factor E4A (UFD2)^c^	Alzayady *et al*. 2005
Ubiquitin fusion degradation 1 protein (UFD1)^c^	Alzayady *et al*. 2005

a Bcl‐2 has also been reported to sensitize IP_3_Rs to IP_3_/Ca^2+^ (see Table 1). ^b^CaBP1 and CIB1 may also cause transient activation of IP_3_Rs, although this is controversial (see Table 1). ^c^Components of the proteasomal pathway.

**Table 3:** Proteins that form complexes with IP_3_Rs and act as downstream effectors

Protein	References
Anoctamin‐1 (ANO1, Ca^2+^‐activated Cl^−^ channel)	Jin *et al*. 2013
Calcineurin (CN; protein phosphatase 2B)	Cameron *et al*. 1995 *a*; Chang *et al*. 2014
CASK (Ca^2+^/calmodulin‐dependent serine protein kinase)	Maximov *et al*. 2003
CRTC2 (CREB‐regulated transcription coactivator 2)	Wang *et al*. 2012
IRBIT (IP_3_‐binding protein released with IP_3_)^a^	Ando *et al*. 2003
KCa1.1 (BK_Ca_; large conductance Ca^2+^‐activated K^+^ channel)	Zhao *et al*. 2010; Mound *et al*. 2013
Na^+^/Ca^2+^ exchanger 1 (NCX1)	Lencesova *et al*. 2004; Mohler *et al*. 2005
Orai‐1 (Ca^2+^ release‐activated Ca^2+^ channel 1)	Woodard *et al*. 2010; Lur *et al*. 2011
Plasma membrane Ca^2+^ ATPase (PMCA)	Shin *et al*. 2000; Huang *et al*. 2006
Protein kinase C (PKC)	Ferris *et al*. 1991; Rex *et al*. 2010
SERCA 2B/3 (sarco/endoplasmic reticulum Ca^2+^‐ATPase)	Redondo *et al*. 2008
STIM1 (stromal interaction molecule 1)	Santoso *et al*. 2011
TRPC1‐7 (transient receptor potential canonical channels)	Boulay *et al*. 1999; Mery *et al*. 2001; Tang *et al*. 2001; Yuan *et al*. 2003; Tong *et al*. 2004
VDAC1 (voltage‐dependent anion channel 1)	Szabadkai *et al*. 2006

a IRBIT also inhibits IP_3_Rs by occluding the IP_3_‐binding site (Table 2).

**Table 4:** Other proteins that form complexes with IP_3_Rs

Protein	References
Cytoskeletal, scaffolding and adaptor proteins	
14‐3‐3 protein zeta/delta (PKC inhibitor protein 1)	Angrand *et al*. 2006
α‐Actin	Sugiyama *et al*. 2000
Ankyrin‐B (ANK2)	Hayashi & Su, 2001; Mohler *et al*. 2004; Kline *et al*. 2008
AKAP9 (A‐kinase anchor protein 9; Yotiao)	Tu *et al*. 2004
BANK1 (B‐cell scaffold protein with ankyrin repeats)	Yokoyama *et al*. 2002
Caveolin‐1	Murata *et al*. 2007; Sundivakkam *et al*. 2009; Jin *et al*. 2013
Coiled‐coil domain‐containing protein 8	Hanson *et al*. 2014
Homer 1/2/3	Tu *et al*. 1998
EB1 / EB3 (end‐binding protein 1/3)^a^	Geyer *et al*. 2015
KRAP (K‐Ras‐induced actin‐interacting protein)	Fujimoto *et al*. 2011
LAT (linker of activated T‐cells)	deSouza *et al*. 2007
Myosin‐2A	Walker *et al*. 2002; Hours & Mery, 2010
Obscurin‐like protein 1	Hanson *et al*. 2014
Protein 4.1N (band 4.1‐like protein 1)	Maximov *et al*. 2003
SEC8 (exocyst complex component)	Shin *et al*. 2000
SNAP‐29 (synaptosomal‐associated protein 29)	Huttlin *et al*. 2013
α‐Spectrin/β‐spectrin (α/β‐fodrin)	Lencesova *et al*. 2004
Syntaxin 1B	Tanaka *et al*. 2011
Talin	Sugiyama *et al*. 2000
Vimentin	Dingli *et al*. 2012
Vinculin	Sugiyama *et al*. 2000
Other proteins	
Anaplastic lymphoma kinase (ALK)	Crockett *et al*. 2004
ARHGAP1 (Rho GTPase‐activating protein 1)	Nagaraja & Kandpal, 2004
γ‐BBH (γ‐butyrobetaine dioxygenase)	Huttlin *et al*. 2013
Beclin‐1	Vicencio *et al*. 2009
BOK (Bcl‐2‐related ovarian killer protein)	Schulman *et al*. 2013
Calnexin	Joseph *et al*. 1999
CD44 antigen (heparin sulphate proteoglycan)	Singleton & Bourguignon, 2004
CEMIP (cell migration‐inducing and hyaluronan‐binding protein)	Tiwari *et al*. 2013
Cyclophilin D (peptidyl‐prolyl cis‐trans isomerase F)	Paillard *et al*. 2013
FAM19A4 (chemokine‐like protein TAFA‐4)	Huttlin *et al*. 2013
F‐box and leucine‐rich repeat protein 14	Huttlin *et al*. 2013
FGL2 (fibrinogen‐like 2)	Huttlin *et al*. 2013
FERM domain‐containing 1	Huttlin *et al*. 2013
GluRδ2 (ionotropic glutamate receptor δ2)	Nakamura *et al*. 2004
Golgi anti‐apoptotic protein (GAAP; Lifeguard 4; TMBIM4)	de Mattia *et al*. 2009
GRP‐75 (glucose‐regulated protein 75; stress‐70 protein)	Szabadkai *et al*. 2006
Heat shock protein 90 (HSP90)	Nguyen *et al*. 2009
Junctate	Treves *et al*. 2004
Lethal(3)malignant brain tumor‐like protein 2	Huttlin *et al*. 2013
Lymphoid‐restricted membrane protein (LRMP; JAW1)	Shindo *et al*. 2010
Na^+^/K^+^‐transporting ATPase	Mohler *et al*. 2005; Yuan *et al*. 2005
Neuronal acetylcholine receptor α3	Huttlin *et al*. 2013
PASK (PAS domain‐containing protein kinase)	Schlafli *et al*. 2011
Phospholamban	Koller *et al*. 2003
Polycystin‐2 (PC2; TRPP2)	Li *et al*. 2005
Protein kinase G1 (PKG1; cGMP‐dependent protein kinase 1)	Schlossman *et al*. 2000
PTPα (protein tyrosine phosphatase‐α)	Wang *et al*. 2009
Rab29 (Ras‐related protein Rab7L1)	Huttlin *et al*. 2013
Rac1 (Ras‐related C3 botulinum toxin substrate 1; TC25)	Natsvlishvili *et al*. 2015
RhoA	Mehta *et al*. 2003
Sigma 1 receptor (σ1R)	Hayashi & Su, 2001; Natsvlishvili *et al*. 2015
Sirtuin‐7	Tsai *et al*. 2012
c‐Src (proto‐oncogene tyrosine‐protein kinase Src)	Jayaraman *et al*. 1996; Wang *et al*. 2009
STARD13 (StAR‐related lipid transfer protein 13; RhoGAP)	Nagaraja & Kandpal, 2004
Syndecan‐1 (SYND1; CD138)	Maximov *et al*. 2003
TESPA1 (thymocyte‐expressed positive selection‐associated protein 1)	Matsuzaki *et al*. 2012

a Both EB1 and EB3 associate with IP_3_Rs, but only EB3 has been shown to be required for effective Ca^2+^ signalling in endothelial cells (Table 1) (Geyer *et al*. 2015).

Although we focus on the ability of IP_3_Rs to release Ca^2+^ from intracellular stores, IP_3_Rs have additional roles. For example, binding of IP_3_ is proposed to release IRBIT from the IP_3_‐binding site, freeing IRBIT to regulate additional targets that include ion channels, transporters and the enzyme ribonucleotide reductase (Ando *et al*. 2014; Arnaoutov & Dasso, 2014). IP_3_Rs may also regulate associated proteins independently of their ability to release Ca^2+^. For example, a direct interaction between IP_3_Rs and TRPC (transient receptor potential canonical) channels is proposed to stimulate opening of the latter (Zhang *et al*. 2001). Hence, when reviewing the effects of proteins associated with IP_3_Rs, we should look beyond the effects of IP_3_ on cytosolic Ca^2+^ signals, to consider also consequences within the ER lumen, effects on Ca^2+^ entry, and effects unrelated to Ca^2+^ signalling. That scope is too ambitious for this short review. Instead we provide a comprehensive summary of proteins suggested to interact with IP_3_Rs (Tables 1, 2, 3, 4, within which we provide most references) and then explore a few selected examples to illustrate some general features.

## Signalling complexes containing IP_3_Rs span entire signalling pathways

The sheer number of proteins reported to form complexes with IP_3_Rs is striking and so too is their diversity, in terms of both cellular geography and function (Tables 1, 2, 3, 4). IP_3_Rs form complexes with many of the proteins that link extracellular stimuli to formation of IP_3_, including G protein‐coupled receptors (GPCRs), the epidermal growth factor receptor (EGFR), the erythropoietin receptor, the Gβγ complexes of G proteins, and some forms of PLC. IP_3_Rs also associate with other signalling proteins linked to PLC signalling, including protein kinase C (PKC), RACK1 (receptor of activated PKC) and the phosphoinositide phosphatase PTEN. The interactions extend also to proteins from other signalling pathways, including adenylyl cyclase (AC), the small G protein K‐Ras, and the protein kinases AKT1 (RAC‐α serine/threonine protein kinase), mTOR (mammalian target of rapamycin), c‐Src and MAPK1/MAPK3 (mitogen‐activated protein kinase 1/3) (Tables 1, 2, 3, 4 and Fig. 1). Proteins that respond to the Ca^2+^ released by IP_3_Rs also form complexes with IP_3_Rs. These include ion channels, exchangers and pumps within the plasma membrane. It is clear that IP_3_Rs reside within macromolecular complexes that both span entire signalling pathways from cell‐surface receptors to the effectors that respond to Ca^2+^, and include proteins that integrate signals from other signalling pathways.

The advantages of these signalling complexes are clear. They allow information to be directed selectively from specific extracellular stimuli to specific intracellular targets through conserved signalling pathways. Furthermore, associated proteins can integrate signals from different signalling pathways and so modulate traffic through the complex. Hence, protein complexes confer both specificity and plasticity. A third advantage is speed. Signalling pathways must be able to turn on and off quickly. Fast activation benefits from high concentrations of reactants and fast on‐rates (*k*_1_) for association of messengers with their targets. Rapid de‐activation requires rapid destruction or dissipation of the messenger and a fast dissociation rate (*k*_−1_). By facilitating delivery of messengers at high local concentrations to their targets (e.g. IP_3_ to IP_3_Rs), signalling complexes contribute to both rapid activation and de‐activation, the latter because diffusion of messengers away from the site of delivery may be sufficient to allow their concentration to fall below that required for activation as soon as synthesis of the messenger ceases. Secondly, targets can have fast off‐rates (*k*_−1_) with a corresponding loss of affinity (equilibrium association constant, *K*_A_ = *k*_1_/*k*_−1_) that does not compromise their capacity to respond to high local concentrations of messenger. We suggest, then, that assembly of proteins around IP_3_Rs contributes to fast and specific signalling, while providing opportunities for signal integration and plasticity.

For convenience, we consider the proteins that associate with IP_3_Rs under four somewhat arbitrary (and overlapping) headings: proteins that enhance or inhibit the activity of IP_3_Rs (Tables 1 and 2); proteins that respond to Ca^2+^ released by IP_3_Rs (Table 3); and proteins with more general roles, including those associated with movement of IP_3_Rs (Table 4).

## Proteins that enhance the function of IP_3_Rs

Usually, IP_3_Rs open only when they have bound both IP_3_ and Ca^2+^ (Foskett *et al*. 2007; Taylor & Tovey, 2010). Unsurprisingly, therefore, most of the proteins that associate with IP_3_Rs and enhance their activity do so either by allowing more effective delivery of IP_3_ and/or Ca^2+^ to IP_3_Rs, or by enhancing the responsiveness of IP_3_Rs to IP_3_ and/or Ca^2+^ (Table 1).

The association of IP_3_Rs with GPCRs, EGFR and erythropoietin receptors, with the βγ subunits of G proteins, with some isoforms of PLC, and with scaffold proteins, like Homer 1 that tethers IP_3_Rs to metabotropic glutamate receptors and PLC (Tu *et al*. 1998), suggest mechanisms by which receptors may effectively deliver IP_3_ to specific IP_3_Rs. This targeted delivery of IP_3_ provides two advantages: it allows rapid responses and it may allow spatially organized Ca^2+^ signals to retain an ‘imprint’ of the stimulus that evoked them. Bradykinin B_2_ receptors (B_2_Rs) are a well‐defined example. In sympathetic neurons, both muscarinic M_1_ receptors (M_1_Rs) and B_2_Rs activate PLC, but only activation of B_2_Rs evokes Ca^2+^ release through IP_3_Rs (Delmas *et al*. 2002). This selectivity arises because B_2_Rs, but not M_1_Rs, form complexes with IP_3_Rs. Rapid generation of IP_3_ in response to activation of B_2_Rs thereby generates relatively high concentrations of IP_3_ in the vicinity of IP_3_Rs, which are not achieved by the more distant M_1_Rs. In this case, selective coupling between plasma membrane receptors and IP_3_Rs may allow sympathetic neurons to generate different intracellular responses to pro‐inflammatory and cholinergic inputs.

Rather than enhancing the delivery of IP_3_ to IP_3_Rs, many other proteins sensitize IP_3_Rs to prevailing concentrations of IP_3_ and/or Ca^2+^ (Table 1). An example, which may play an important role in human disease, is the sensitization of IP_3_Rs by mutant forms of presenilins (Cheung *et al*. 2008). Mutations in presenilin‐1 (PS1) and presenilin‐2 (PS2) are major causes of familial Alzheimer's disease. Although both wild‐type and mutant presenilins associate with IP_3_Rs, only the disease‐causing mutant forms of PS1 and PS2 enhance the activity of IP_3_Rs in response to IP_3_ and Ca^2+^. The mechanism involved may be a change in the modal gating of IP_3_Rs (Cheung *et al*. 2010). This increased activity of IP_3_Rs results in enhanced release of Ca^2+^, which may lead to aberrant processing of β‐amyloid (Cheung *et al*. 2008), constitutive activation of cyclic AMP response element binding protein (CREB)‐mediated transcription (Muller *et al*. 2011), synaptic dysfunction and neuronal degeneration (Mattson, 2010).

Although activation of IP_3_Rs normally requires binding of IP_3_ and Ca^2+^, a few proteins have been reported to cause reversible activation of IP_3_Rs directly, without the coincident presence of IP_3_ and Ca^2+^ (Table 1). These include Gβγ (Zeng *et al*. 2003), CIB1 (White *et al*. 2006) and, more controversially, CaBP1 (Yang *et al*. 2002). The initial report on the actions of CaBP1 described an activation of *Xenopus* IP_3_Rs in the absence of IP_3_ *in vitro*. However, subsequent studies have demonstrated that CaBP1 inhibits Ca^2+^ release via mammalian and *Xenopus* IP_3_Rs by stabilizing an inactive state of the IP_3_R (Haynes *et al*. 2004; Nadif Kasri *et al*. 2004; White *et al*. 2006; Li *et al*. 2013). Similarly, CIB1 was reported to activate IP_3_Rs in *Xenopus* oocytes and Sf9 insect cells in the absence of IP_3_, but it too inhibits Ca^2+^ release via mammalian IP_3_Rs (White *et al*. 2006). Uniquely, an irreversible activation of IP_3_Rs appears to occur after proteolytic cleavage by caspase‐3 (Assefa *et al*. 2004; Nakayama *et al*. 2004), a process that may play a prominent role in apoptosis.

## Proteins that inhibit the function of IP_3_Rs

Many proteins that interact with IP_3_Rs inhibit their function (Table 2). These interactions may enable rapid feedback regulation of Ca^2+^ release and provide long‐term attenuation of IP_3_R activity by promoting degradation or irreversible inhibition of IP_3_Rs. These mechanisms contribute to the tight regulation of IP_3_R activity needed to achieve spatial and temporal organization of Ca^2+^ signals (Konieczny *et al*. 2012). They also provide protection from the damaging consequences of excessive increases in cytosolic free Ca^2+^ concentration (Orrenius *et al*. 2015) and disturbance of the other essential roles of the ER while it fulfils its role in Ca^2+^ signalling (Berridge, 2002). Proteins that inhibit IP_3_Rs in a Ca^2+^‐dependent manner, like calmodulin, CaBP1, calcineurin, CaMKII and the unidentified protein(s) that may mediate the universal inhibition of IP_3_Rs by Ca^2+^, are prime candidates for mediating this negative feedback. Proteins that inhibit IP_3_Rs fall into two broad categories: those that bind reversibly to interfere with binding of IP_3_ and/or Ca^2+^ or their links to gating; and those that cause post‐translational modifications of the IP_3_R (Table 2).

**Figure 2:**
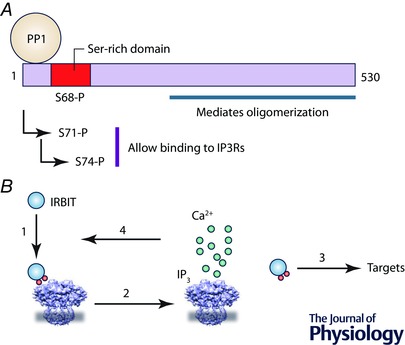
**IRBIT controls the sensitivity of IP_3_Rs** *A*, the N‐terminal region of IRBIT includes a serine‐rich domain. Phosphorylation of S68, the ‘master’ phosphorylation site, allows sequential phosphorylation of the two residues, S71 and S74, that must be phosphorylated for IRBIT to bind to IP_3_Rs. Protein phosphatase 1 (PP1) bound to IRBIT dephosphorylates S68. *B*, phosphorylation of IRBIT (1) allows it to bind to the IBC and so compete with IP_3_ for binding to the IP_3_R. Phospho‐IRBIT thereby sets the sensitivity of the IP_3_R to IP_3_. IP_3_ binding to the IBC (2) prevents IRBIT binding and initiates activation of the IP_3_R. The displaced phospho‐IRBIT can regulate many additional targets, including ion channels and transporters (3). The Ca^2+^ released by active IP_3_Rs may control the phosphorylation state of IRBIT, and thereby complete a feedback loop that regulates IP_3_R sensitivity (4).

IRBIT inhibits all three IP_3_R subtypes by competing with IP_3_ for binding to the IBC (Ando *et al*. 2003). IRBIT binds only when it is phosphorylated at several sites, probably because the phosphorylated residues mimic the essential phosphate groups of IP_3_ (Fig. 2 *A*). Residue S68 is the ‘master’ phosphorylation site. When it is phosphorylated by a Ca^2+^‐dependent kinase, perhaps a Ca^2+^/calmodulin‐dependent protein kinase (CaMK), it allows casein kinase I‐mediated phosphorylation of the two residues (S71 and S74, residue numbering relates to mouse IP_3_R1) that are critical for binding of IRBIT to IP_3_Rs (and its other targets) (Ando *et al*. 2014). Dephosphorylation of S68 is catalysed by protein phosphatase 1 (PP1), which also associates with IRBIT. The competition between phospho‐IRBIT and IP_3_ for occupancy of the IBC through which IP_3_ initiates activation of IP_3_Rs allows IRBIT to tune the sensitivity of IP_3_Rs to IP_3_. Hence, inhibiting expression of IRBIT, or expression of a dominant negative form (IRBIT‐S68A), allows Ca^2+^ release at lower concentrations of IP_3_ (Ando *et al*. 2014). This tuning of IP_3_R sensitivity has been demonstrated in sympathetic neurons where, as discussed earlier, M_1_Rs do not associate with IP_3_Rs and do not normally generate sufficient IP_3_ to activate more distant IP_3_Rs (Delmas *et al*. 2002). However, expression of the dominant negative IRBIT allows M_1_Rs to evoke Ca^2+^ release through IP_3_Rs (Zaika *et al*. 2011). Although the details are not fully resolved, the interplay between Ca^2+^ and the activation of IRBIT is intriguing because it suggests potential feedback loops that might control the sensitivity of IP_3_Rs to IP_3_ (Ando *et al*. 2014). The phosphorylation (of S68) that initiates activation of IRBIT is Ca^2+^ sensitive, deactivation of IRBIT by proteolytic cleavage within its N‐terminal may be mediated by Ca^2+^‐sensitive calpain, and IRBIT itself inhibits Ca^2+^/calmodulin‐dependent protein kinase IIα (CaMKIIα) (Kawaai *et al*. 2015) (Fig. 2 *B*).

Post‐translational modification of IP_3_Rs by associated proteins may be reversible (e.g. phosphorylation) (Betzenhauser & Yule, 2010) or irreversible (e.g. proteolysis and some covalent modifications). An example of the latter is the Ca^2+^‐dependent enzyme transglutaminase type 2 (TGM2). By covalently modifying a glutamine residue within the C‐terminal tail of IP_3_R1, TGM2 causes irreversible cross‐linking of adjacent IP_3_R subunits via a lysine residue and the modified glutamine. This prevents the conformational changes required for activation of IP_3_Rs, and so inhibits IP_3_‐evoked Ca^2+^ release (Hamada *et al*. 2014). The Ca^2+^ sensitivity of TGM2 may allow it to contribute to feedback control of Ca^2+^ release and to disruption of IP_3_R function when dysregulation of Ca^2+^ signalling occurs in pathological conditions such as Huntington's disease (Hamada *et al*. 2014). Activation of IP_3_Rs and the ensuing release of Ca^2+^ also trigger ubiquitination and proteasomal degradation of IP_3_Rs (Pearce *et al*. 2009) and their cleavage by calpains (Μagnusson *et al*. 1993; Wojcikiewicz & Oberdorf, 1996). Hence, proteins that associate with IP_3_Rs provide mechanisms that allow both acute and long‐term feedback regulation of IP_3_R activity.

## Downstream effectors

IP_3_Rs also form complexes with proteins that are downstream effectors of IP_3_R activation; most of these respond to the Ca^2+^ released by IP_3_Rs (Table 3). Many of these proteins are cytosolic, but others reside within membranes that allow IP_3_Rs within the ER to communicate with other intracellular organelles or the plasma membrane. The importance of this communication between organelles, mediated by junctional complexes between them, is increasingly recognized (Lam & Galione, 2013).

**Figure 3:**
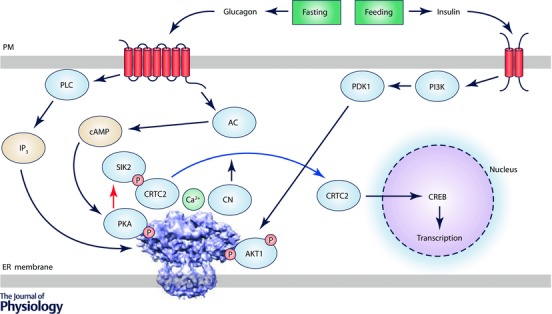
**A signalling complex assembled around IP_3_Rs controls gluconeogenesis** Glucagon and insulin exert opposing effects on hepatic gluconeogenesis. Their signalling pathways converge to a protein complex assembled around IP_3_Rs, the activity of which controls phosphorylation of the transcription factor CRTC2. Dephosphorylated CRTC2 translocates to the nucleus, where it associates with CREB and stimulates transcription of genes required for gluconeogenesis. SIK2 phosphorylates CRTC2, while calcineurin dephosphorylates it. Glucagon, via a GPCR, stimulates both PLC and AC. The IP_3_ produced by PLC stimulates IP_3_Rs. The cAMP generated by AC stimulates PKA and that promotes dephosphorylation of CRTC2 by phosphorylating both SIK2 (inhibiting its activity) and IP_3_Rs, sensitizing the latter to IP_3_. The larger Ca^2+^ signal then activates calcineurin. Insulin causes activation of AKT1, which phosphorylates IP_3_Rs and inhibits their activity; it thereby opposes the effects of glucagon and attenuates calcineurin activity. Phosphorylation is indicated by red circles, black arrows denote stimulation and the red arrow denotes inhibition. Abbreviations and further details in the text and tables.

Hepatic gluconeogenesis, which is likely to play an important role in diabetes and obesity, is stimulated by glucagon released by the pancreas during fasting, and inhibited by insulin released when the plasma glucose concentration increases. A complex containing IP_3_Rs, the Ca^2+^‐regulated protein phosphatase calcineurin, the transcriptional co‐activator of CREB‐regulated transcription CRTC2 (CREB‐coactivator C2), PKA and AKT1 coordinates gluconeogenesis (Wang *et al*. 2012) (Fig. 3). De‐phosphorylated CRTC2 binds to nuclear CREB and up‐regulates genes that promote gluconeogenesis. This is repressed by SIK2, a kinase that phosphorylates CRTC2. IP_3_‐evoked Ca^2+^ release activates calcineurin, which de‐phosphorylates CRTC2. Glucagon receptors stimulate production of both cAMP and IP_3_ (Wakelam *et al*. 1986; Wang *et al*. 2012). The cAMP activates PKA, which phosphorylates, and thereby inhibits, SIK2; and it phosphorylates IP_3_Rs, sensitizing them to activation by IP_3_ and Ca^2+^. IP_3_Rs are also directly sensitized by cAMP (Tovey *et al*. 2008). Increased release of Ca^2+^ via IP_3_Rs activates calcineurin, which dephosphorylates CRTC2 (Vanderheyden *et al*. 2009 *a*; Wang *et al*. 2012). Hence glucagon both inhibits the kinase (SIK2) and stimulates the phosphatase (calcineurin) that control phosphorylation of CRTC2. Glucagon also reduces binding of CRTC2 to IP_3_Rs (Wang *et al*. 2012), further enhancing the nuclear translocation of dephosphorylated CRTC2. The signals evoked by insulin receptors also feed into this IP_3_R complex. Insulin stimulates phosphatidylinositol 3‐kinase (PI3K) and thereby AKT1. The latter phosphorylates IP_3_Rs and attenuates their activity. Hence insulin, by inhibiting IP_3_Rs, opposes the actions of glucagon by restraining the activation of calcineurin and so maintains CRTC2 in its inactive phosphorylated state (Wang *et al*. 2012). This example illustrates some of the intricate interactions that the assembly of proteins around IP_3_Rs can allow: signals from a GPCR and a receptor tyrosine kinase converge at IP_3_Rs, which then integrate the inputs and transduce them into a regulation of gene expression (Fig. 3).

## Proteins that determine the distribution of IP_3_Rs

The subcellular distribution of IP_3_Rs is an important influence on their behaviour, not least because it defines the sites at which they will release Ca^2+^, and whether they will be exposed to effective concentrations of the stimuli that activate them, IP_3_ and Ca^2+^. Assembly of IP_3_Rs with components of the PLC signalling pathway (see above) can ensure targeted delivery of IP_3_, but Ca^2+^ is most often provided by neighbouring IP_3_Rs. An important interaction, therefore, is that between IP_3_Rs themselves, because their proximity to neighbours dictates whether Ca^2+^ released by an active IP_3_R can ignite the activity of other IP_3_Rs. Considerable evidence suggests that clustering of IP_3_Rs within the plane of the ER membrane is dynamically regulated by IP_3_ and/or Ca^2+^ (Tateishi *et al*. 2005; Rahman *et al*. 2009; and see references in Geyer *et al*. 2015), although the role of this process in shaping Ca^2+^ signals remains controversial (Smith *et al*. 2014). We have suggested that IP_3_‐evoked clustering of IP_3_Rs may contribute to the coordinated openings of IP_3_Rs that underlie the small Ca^2+^ signals (‘Ca^2+^ puffs’) evoked by low stimulus intensities, by both bringing IP_3_Rs together and retuning their Ca^2+^ sensitivity (Rahman *et al*. 2009). Head‐to‐head interactions of IP_3_Rs have also been observed in electron micrographs of purified IP_3_Rs (Hamada *et al*. 2003), between opposing ER membranes within cells (Takei *et al*. 1994) and between the isolated N‐terminal domains of IP_3_Rs (Chavda *et al*. 2013). The functional significance of these interactions has not been established.

**Figure 4:**
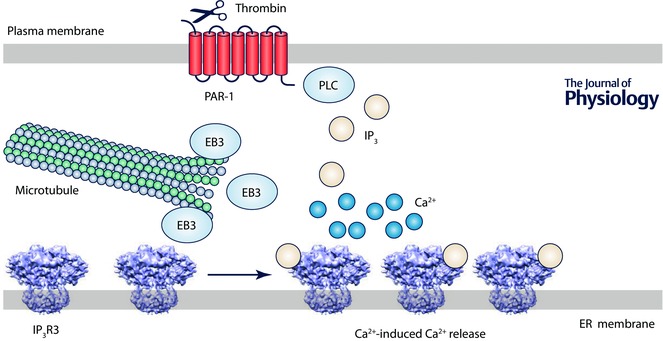
**EB3 is required for effective signalling by IP_3_Rs in endothelial cells** In endothelial cells, EB3 binds to a TxIP motif within the regulatory domain of IP_3_R3, allowing IP_3_Rs to associate with the plus‐end of microtubules. Disrupting this interaction prevents clustering of IP_3_Rs and attenuates the Ca^2+^ signals evoked by thrombin, which cleaves within the N‐terminus of PAR‐1 and allows it to stimulate PLC. The evidence (Geyer *et al*. 2015) suggests that the EB3‐mediated interaction of IP_3_R3 with microtubules is essential for the clustering of IP_3_Rs that allows the Ca^2+^ released by one IP_3_R to be amplified by recruitment of neighbouring IP_3_Rs.

A recent study of the Ca^2+^ signals evoked by thrombin‐mediated stimulation of the protease‐activated receptor PAR‐1 in endothelial cells provides evidence that microtubules may guide IP_3_Rs into the clusters within which Ca^2+^ release can most effectively recruit neighbouring IP_3_Rs (Geyer *et al*. 2015). In lung microvascular endothelial cells, thrombin, which activates PAR‐1 by cleaving its N‐terminal, stimulates PLC and thereby evokes Ca^2+^ release through IP_3_Rs. The resulting increase in cytosolic Ca^2+^ concentration contributes to disassembly of the adherens junctions that maintain the integrity of the endothelium (Komarova & Malik, 2010). These effects are attenuated when the interaction between type 3 IP_3_Rs (IP_3_R3) and end‐binding protein 3 (EB3) are disrupted. EB3 belongs to a family of proteins that bind to the plus‐end of growing microtubules and recruit other proteins, often via an S/TxIP motif (where x denotes any residue) (Honnappa *et al*. 2009). Mutation of the TxIP motif within the regulatory domain of IP_3_R3 prevents its binding to EB3, attenuates thrombin‐evoked Ca^2+^ signals, and reduces both the basal clustering of IP_3_R3 and the enhanced clustering evoked by thrombin. Hence, in endothelial cells, the association of IP_3_R3 with EB3 and microtubules is required for both clustering of IP_3_Rs and effective Ca^2+^ signalling. This suggests that clustering allows IP_3_Rs to deliver Ca^2+^ more effectively to other IP_3_Rs and so allows the amplification provided by Ca^2+^‐induced Ca^2+^ release (Fig. 4). We conclude that association of IP_3_Rs with other proteins, components of the PLC signalling pathway or EB3, contributes to effective delivery of the two essential regulators of IP_3_Rs, IP_3_ and Ca^2+^, respectively.

## Conclusions

IP_3_Rs and the Ca^2+^ they release are called upon to specifically regulate many physiological processes (Berridge, 2009), while neither perturbing the other essential roles of the ER (Berridge, 2002) nor subjecting the cell to the deleterious consequences of excessive increases in cytosolic Ca^2+^ concentration (Orrenius *et al*. 2015). These demands impose a need for complex regulation of IP_3_Rs, much of which is achieved by assembling proteins around IP_3_Rs to form signalling complexes (Konieczny *et al*. 2012). These complexes allow signals to be directed through conserved signalling pathways and endow the pathways with speed, integrative capacity and plasticity. The very large size of IP_3_Rs relative to most other ion channels might be viewed as an evolutionary adaptation to meet this need for them to function as signalling hubs.

Advances in genomics, proteomics, antibody technologies and bioinformatics have transformed analyses of protein–protein interactions. It is now possible to interrogate these interactions on a whole‐proteome scale (Havugimana *et al*. 2012; Rolland *et al*. 2014). Bioinformatic methods can predict protein–protein interactions (Baughman *et al*. 2011; Kotlyar *et al*. 2015) and even the regions of the proteins that are involved (Gavenonis *et al*. 2014). These powerful technologies, and the opportunities they provide to design new therapies (Wells & McClendon, 2007), cannot displace the need for direct confirmation of the interactions and their functional significance. Together, these approaches pave the way to defining the properties and functional importance of IP_3_R signalling hubs in normal physiology and disease.

## Additional information

### Competing interests

None declared.

### Author contributions

All authors have approved the final version of the manuscript and agree to be accountable for all aspects of the work. All persons designated as authors qualify for authorship, and all those who qualify for authorship are listed.

### Funding

This work was supported by the Biotechnology and Biological Sciences Research Council (L0000075) and the Wellcome Trust (101844).
